# Patient-specific depth of endotracheal intubation-from anthropometry to the Touch and Read Method

**DOI:** 10.12669/pjms.325.10609

**Published:** 2016

**Authors:** Saecheol Oh, Seunguk Bang, Woojin Kwon, Jungwoo Shim

**Affiliations:** 1 Dr., Saecheol Oh, MD, PhD. Department of Anaesthesiology and Pain Medicine College of Medicine, The Catholic University of Korea Seoul, Korea; 2 Dr. Seunguk Bang, MD, PhD. Department of Anaesthesiology and Pain Medicine College of Medicine, The Catholic University of Korea Seoul, Korea; 3 Dr. Woojin Kwon, MD. Department of Anaesthesiology and Pain Medicine College of Medicine, The Catholic University of Korea Seoul, Korea; 4 Dr. Jungwoo Shim, MD. Department of Anaesthesiology and Pain Medicine College of Medicine, The Catholic University of Korea Seoul, Korea

**Keywords:** Airway, Complications, intubation, Laryngoscopy, Safety

## Abstract

**Objective:**

Knowledge of accurate airway length (AL) enables safer placement of the endotracheal tube (ETT) in the trachea. Our objective was to check the safety of a new formula (Touch and Read method) to determine ETT depth.

**Methods:**

AL was measured in 176 patients. Patients were divided into a normal group (AL >25 cm in men, >23 cm in women) and a risk group (AL ≤25 cm in men, ≤23cm in women). A control test (Conventional method) was performed in which the ETT was secured at a depth of 23 cm from the central incisor in men and 21 cm in women. In the experimental test (Touch and Read method), the ETT was secured at a depth equal to the distance from the angle of the mouth to the epiglottis tip plus 12.5 cm in men and 11.5 cm in women. The mean distance from the tube tip to the carina and that from the vocal cords to tube cuff were compared between the control and experimental tests in each group.

**Results:**

The two distances were similar between control and experimental tests in the normal group, but differed in the risk group (Women: mean distance from tube tip to carina, 1.2 cm and from vocal cords to cuff, 2.7 cm [control test]; 1.9 and 2.0 cm, respectively [experimental test]. Men: 0.7 and 3.5 cm, respectively [control test]; 2.0 and 2.3 cm, respectively [experimental test]).

**Conclusion:**

Touch and Read method enables safer placement of the ETT in the trachea than the conventional method in the risk group.

## INTRODUCTION

In patients whose vocal cords are completely visible with direct laryngoscopy (Cormack-Lehane grade I), it is a standard rule that anaesthesiologists place the endotracheal tube with its proximal cuff to be positioned 2 cm below the vocal cords.^1^ Around 73% of patients belong to this group. However, the challenge is how to determine the depth of intubation in the remainder (27%) of patients whose vocal cords are partially visible or invisible (Cormack-Lehane grade II or higher).^2^ The depth of endotracheal intubation using the conventional method is 23 cm from the central incisor in men, and 21 cm in women.^3^ These standard depths were derived using data from patients whose height was 168–184 cm (men) and 158–174 cm (women). There is no clear recommendation for patients with heights outside these ranges.

In this study, we measured the airway length from the central incisor to the carina. On the basis of these measurements, we developed a new method to determine patient-specific depth of endotracheal intubation. We have also performed a virtual experiment to evaluate the efficacy of the experimental versus the conventional method in clinical practice on the basis of airway data.

Our objective was to check the safety of a new formula (Touch and Read method) to determine ETT depth.

## METHODS

Approval of the Institutional Review Board (IRB # DC11OISI0068) of the Catholic University was obtained prior to enrolment of patients in the study, and written informed consent was obtained from all patients. The study was registered at ClinicalTrials.gov (NCT02158247).

The study included patients aged over 20 years, with American Society of Anaesthesiologists (ASA) physical status class I and II, who were enrolled from September 2010 to January 2012. We excluded patients who received general anaesthesia with face mask ventilation or laryngeal mask airway and those who received videolaryngoscope- or fiberoptic bronchoscope-assisted intubation.

A total of 181 patients initially met the inclusion criteria. However, five patients (three women and two men) were excluded from the analysis because of the lack of visible vocal cords, even with a videolaryngoscope. A total of 69 male and 107 female patients were included in the final analysis.

**Fig.1:**
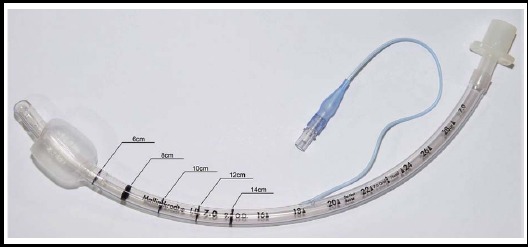
7-mm inner diameter endotracheal tube that has been scaled in 1-cm increments from the distal end for use in our study.

Endotracheal intubation was performed with a 7.0-mm inner diameter (I.D.) endotracheal tube (Hi-Lo™, Mallinckrodt, Ireland). It was artificially calibrated via marking with oil-based ink from the distal tip of the tube at 1-cm increments. A point “P”, 2 cm from the proximal end of the cuff and 8 cm from the distal tip of the tube, was marked on the tube (Fig.1).

### Getting the raw airway data

The whole length of the endotracheal tube, T, was measured with a flexible measuring tape. General anaesthesia was induced under standard anaesthetic monitoring using propofol (1–2.5 mg/kg) and rocuronium (0.5 mg/kg). After sufficient pre-oxygenation with 100% oxygen, the anaesthesiologist opened the patient’s mouth wide, and the MacIntosh laryngoscope blade (#3; Classic+, Macintosh Fiber Optic^®^, HEINE, Germany) was introduced. The endotracheal tube was advanced to the epiglottis tip with maximal exposure of the vocal cords. On touching the tip of the epiglottis with the endotracheal tube, the depth of the tube was measured by reading the scale on the tube at the angle of the mouth. This was the length of the airway from the angle of the mouth to the epiglottis tip (A_1_).

After withdrawal of the laryngoscope and endotracheal tube, the videolaryngoscope (Glidescope^®^ videolaryngoscope, Verathon Inc., Bothell, WA, USA) was introduced to guide the placement of the endotracheal tube, so that the point P (2 cm above the cuff of the tube) on the tube was at the level of the vocal cords. The cuff was then inflated and the videolaryngoscope was withdrawn. The tube depth from the angle of the mouth was noted as B. The tube was then repositioned to the patient’s midline to read the depth of the endotracheal tube at the central incisor. This depth was noted as C.

**Fig.2:**
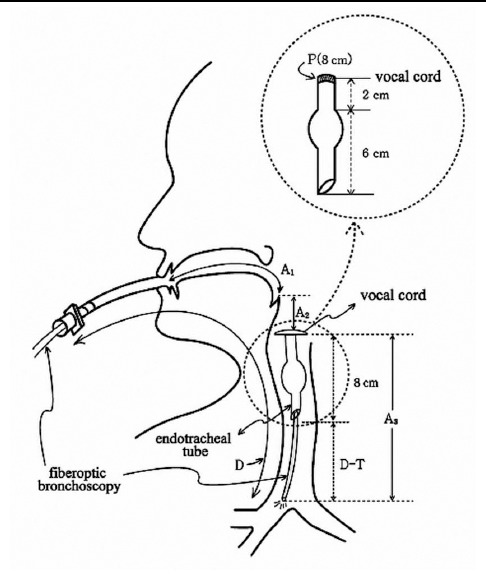
Schematic diagram illustrating the various points that were measured along the length of the airway length and of the calculated distances.

Finally, the endotracheal tube was secured at the depth B on the angle of the mouth of the patient. While maintaining anaesthesia with a mixture of O_2_ and an inhalational anaesthetic agent, a fiberoptic bronchoscope (Olympus BF type 3C40, Olympus Optical Co., Japan) was passed through the endotracheal tube. Keeping the centre of the bronchus and the bronchoscopic view at the same level, the bronchoscope was advanced to the carina. The bronchoscope was turned to the right main bronchus and advanced until its circular entrance disappeared from the view. The point D was marked on the bronchoscope at the proximal end of the tube. D represented the length from the carina to the proximal end of the tube (Fig.2). The airway length calculations are described in Table-I.

**Table-I:** Summary of airway measurements; calculation and equations.

*Points and Measurements*		
A_1_	Distance from the mouth angle to the tip of epiglottis	
A_2_	Distance from the epiglottis tip to the vocal cords	
A_3_	Distance from the vocal cords to the carina	
B	Final depth of intubation measured from the mouth angle	
C	Final depth of intubation from the central incisor	
D	Distance from the carina to the proximal end of tube	
I	C – B	
T	Full length of the endotracheal tube	
*Calculation of distances based on points*		
From the mouth angle to the carina		A = A_1_ + A_2_ + A_3_
From the mouth angle to tip of the epiglottis		A_1_
From the epiglottis tip to the vocal cords		A_2_ = B − 8 − A_1_
From the vocal cords to the carina		A_3_ = D − T + 8 *
From the central incisor to the carina		A + I
*Equations for two safety parameters in two methods of intubation by virtual experiment*		
*Tube Tip to Carina*		
Touch and Read Method		Men = A − (A_1_ + 12.5)
		Women = A − (A_1_ + 11.5)
Conventional Method		Men = (A + I) – 23
		Women = (A + I) − 21
*Vocal cords to cuff*		
Touch and Read Method		Men = A_3_ − (D - T + 6)
		Women = A_3_ − (D - T + 6)
Conventional Method		Men = A_3_ − (D - T + 6)
		Women = A_3_ − (D - T + 6)

* The constant, 8, is 8 cm from the distal tip of the tube, which also corresponds to the point that is 2 cm proximal from the proximal end of the cuff in the 7.0 mm inner diameter endotracheal tube.

### Conventional method vs. Touch and Read method

The virtual experiment was performed using the airway database.

### Conventional method

The endotracheal tube was placed at 23 cm or 21 cm from the central incisor for male and female patients, respectively.

### Touch and Read method

The Touch and Read method was made to place the cuff of endotracheal tube 2 cm below the vocal cords with the help of anthropometric data in the human upper airway geometry. The formula for the Touch and Read method is as follows:

Depth of intubation by Touch and Read method = A_1_ (from mouth angle to epiglottis tip) + A_2_ (from epiglottis tip to vocal cords) + 8cm (constant and equal to “P”, from the vocal cords to the tube tip)

Considering that the mean A_2_ is 4.4 cm for men and 3.6 cm for women, the formula would be changed as follows:

Depth = A_1_ + 12.4 cm for men, and A_1_ + 11.6 cm for women.

If we allow for the approximation of the constant 12.4 as 12.5, and 11.6 as 11.5, the final depth of endotracheal tube will be A_1_ + 12.5 cm for men and A_1_ + 11.5 cm for women.

### Virtual test to compare two parameters of safety margin between two methods

Patients were divided into normal and the risk groups. Patients whose airway length from the central incisor to the carina >25 cm (men) or >23 cm (women) were assigned to the normal group, while those whose airway length was ≤25 cm (men) and ≤23 cm (women) were assigned to the risk group. The two different methods of intubation were simulated for each patient.

We calculated two parameters to determine where the tip of the endotracheal tube would be placed if the depth of the endotracheal tube was measured using the conventional or the experimental method: (1) the distance from the tube tip to the carina, and (2) the distance from the vocal cords to the tube cuff. The parameters were calculated mathematically from the airway data of the individual patients. There are equations for two safety parameters in Table-I.

### Statistical analysis

From the pilot study, the standard deviations of the distance from the tube tip to the carina by conventional and experimental (Touch and Read) methods were calculated as σ_1_ = 1.8 cm and σ_2_ = 1.6 cm, respectively. Assuming a 1 cm difference between two means of two tests as clinically significant, the sample size was calculated with a power = 80%, and significance level = 5%. The minimum sample size calculated was 58. To account for potential dropouts, the sample size was set at 70 for the experiment. Considering anthropometric differences between men and women, the sample size was doubled to 140.

The comparison of height and age with airway length (AL) was performed using simple linear regression. Comparison of the AL between male and female patients was performed using an unpaired t-test. The Wilcoxon signed-ranks test was used for the comparing the safety margins between the conventional and the touch and read methods in normal and risk groups. Statistical analysis was performed using SPSS 20.0 (Chicago, IL, USA). Statistical significance was considered as p<0.05.

## RESULTS

All airway lengths were statistically different between men and women (Table-II). The correlation of the patient’s height and age with their airway length was analysed using simple linear regression (Table-III). And the virtual test demonstrated no significant differences in the two parameters between the two methods in the normal group. However, there were significant differences in the risk group (Table-IV).

**Table-II:** Patient characteristics and airway measurements.

*Patients (n=176)*	*Age (years)*	*Height (cm)*	*CI –Carina (cm)*	*CI – MA (cm)*	*MA –Carina (cm)*	*MA –Epiglottis (cm)*	*Epiglottis – VC (cm)*	*VC –Carina (cm)*
Men (n=69)	49.5±13.6	168.6±7.4	26.4±1.9	0.9±0.5	25.5±1.8	9.1±1.0	4.4±0.9	11.9±1.3
Women (n=107)	55.7±11.7	156.4±5.2	24.2±1.5	1.1±0.5	23.2±1.5	8.3±1.0	3.6±0.9	11.2±1.2
p-value	<0.01	<0.01	<0.001	<0.05	<0.001	<0.001	<0.001	<0.001

CI: central incisor, MA: mouth angle, VC: vocal cord.

**Table-III:** Correlations of height and age against airway length.

	*Men (P)*	*Women (P)*
*Correlation between Height and Airway Length*		
Mouth Angle - Carina	< 0.01	< 0.01
Mouth Angle - Epiglottis	< 0.01	< 0.05
Epiglottis-Vocal Cord	> 0.05	> 0.05
Vocal Cord - Carina	< 0.01	< 0.01
*Correlation between Age and Airway Length*		
Mouth Angle - Carina	< 0.01	> 0.05
Mouth Angle - Epiglottis	< 0.05	< 0.05
Epiglottis-Vocal Cord	> 0.05	> 0.05
Vocal Cord - Carina	> 0.05	> 0.05

P < 0.05 indicates statistically significant correlation.

**Table-IV:** Comparison of two safety parameters in two different methods.

	*Tube tip–Carina (cm)*			*VC–Cuff (cm)*		

	*Touch and Read Method*	*Conventional Method*	*P-value*	*Touch and Read Method*	*Conventional Method*	*P-value*
*Normal Group*						
Men (n=54)	4.4 ± 1.2	4.1 ± 1.2	0.08	2.0 ± 1.0	2.3 ± 0.8	0.08
Women (n=83)	3.8 ± 1.0	3.8 ± 1.0	0.69	1.9 ± 1.0	1.8 ± 0.9	0.69
*Risk Group*						
Men (n=15)	2.0 ± 1.2	0.7 ± 1.4	< 0.01	2.3 ± 1.1	3.5 ± 1.4	< 0.01
Women (n=24)	1.9 ± 1.1	1.2 ± 1.0	< 0.01	2.0 ± 0.9	2.7 ± 0.9	< 0.01

Values are mean (SD). Tube tip–Carina: distance from the distal tip of the tube to the carina, VC–Cuff: distance from the vocal cords to the tube cuff.

## DISCUSSION

The study showed that the risk of endobronchial intubation is increased in patients whose airway length is relatively short if we determine the depth of intubation using the conventional method.

There exists a controversy about determining the depth of intubation using the conventional method.^4^,^5^ Sitzwohl et al.^6^ suggested that the depth of intubation by the conventional method is too deep. They recommended the depth of intubation should be 22 cm from the central incisor for men and 20 cm for women instead of 23 cm and 21 cm for men and women, respectively. Moreover, Park et al.^7^ reported that the safety length for endotracheal tube fixation is 20 cm in Korean adult males and 18 cm in Korean adult females.

Additionally, previous studies have shown that endotracheal tube displacement can be the result of head and neck movement.^8^-^14^ As laparoscopic surgery becomes a popular technique, more surgical patients will be placed in the Trendelenburg position, creating positive pressure inflation in the peritoneal space. This is one of the risk factors for endotracheal tube to advance to the bronchus.^15^

Our results clarify the use of depth of intubation by the conventional method. There were no problems using the conventional method in patients with sufficient airway length. However, endotracheal tubes were placed deeper in the tracheas of the patients with relatively short airway length.

Predicting the short airway length avoids the problem of positioning the endotracheal tube too deep using the conventional method. Unfortunately, it is not easy to predict the airway length of the patients. Our results demonstrated significant correlation of airway length with height. However, the correlation coefficient is 0.38 in men and 0.22 in women, which indicates that the height of the patient is not sufficient as a single marker to anticipate airway length. This is why we based the patient-specific depth of intubation on the anthropometry of the human airway, rather than on the predetermined depth by the conventional method.

Although our airway length was calculated from indirect measurement, it is consistent with previous airway data. Park et al.^16^ reported airway length from the central incisor to the carina, measured using a flexible fiberoptic bronchoscope, to be 27.1 cm for men and 25.1 cm for women in patients under general anaesthesia. Our results were 26.4 cm for men and 24.2 cm for women. On the other hand, other studies measured the distance from the vocal cords to the carina to be from 13.0 cm to 13.1 cm for men and from 11.2 cm to 11.9 cm for women, using computerized tomography (CT) and fiberoptic bronchoscope.^17^,^18^ Our results were 11.9 cm and 11.2 cm, respectively. Our method demonstrated comparable consistency of data, despite of different way of airway length measurement.

Although airway length from the angle of the mouth to the carina was significantly correlated to the height of the patients, the length from the epiglottis tip to the vocal cords neither correlated with height nor with age. The length from the epiglottis tip to the vocal cords is relatively constant regardless of the height and age of the patients. It is reasonable for us to consider that as a constant. The mean length is 4.4 cm for men and 3.6 cm for women. This provides the theoretical background for determining the depth of endotracheal tube by Touch and Read method.

We conducted a virtual test to see if there is any clinical advantage of endotracheal intubation by Touch and Read method compared to the conventional method. We enrolled the patients whose airway length from the central incisor to the carina was less than 25 cm in men, and 23 cm in women in the risk group. The cut off was made considering that the endotracheal tube may slide around 1.9 cm with the flexion and extension of the neck and the depths of intubation made by conventional method are 23 cm for men and 21 cm for women.^19^

There were no significant differences in the two parameters of safety margins between the conventional and Touch and Read method in the normal group. However, there were significant differences in the risk group. The mean safety margin from the tube tip to the carina was 1.3 cm longer for men and 0.7 cm longer for women by the Touch and Read method compared to the conventional method. Furthermore, the distance from the vocal cords to the tube cuff was 1.2 cm longer for men and 0.7 cm longer for women. These results imply that the Touch and Read method could be a way to determine the depth of endotracheal intubation.

There are several limitations in this study. First, the airway lengths were measured indirectly or calculated from the formula, except A_1_ (distance from the angle of the mouth to the epiglottis tip). More sophisticated imaging like computerized tomography or magnetic resonance imaging would be needed for accurate measurement of the length of the airway. Second, our test to demonstrate the clinical usefulness of the Touch and Read method was a virtual experiment. A clinical trial is recommended. Third, the constant (11.5 for women and 12.5 for men) is depends on the size or the manufacturer of the endotracheal tube. Fourth, a new endotracheal tube that is scaled numerically from the tip is needed.

In summary, it is reasonable to place the endotracheal tube 2 cm below the vocal cords for the patients whose whole vocal cords are visible. However, if the vocal cords are partially visible or are invisible, Touch and Read method would be a way to determine the depth of the endotracheal intubation for patients whose airway length is supposed to be shorter compared to normal length patients.
